# Facilitating Preemptive Hardware System Design Using Partial Reconfiguration Techniques

**DOI:** 10.1155/2014/164059

**Published:** 2014-02-06

**Authors:** Julio Dondo Gazzano, Fernando Rincon, Carlos Vaderrama, Felix Villanueva, Julian Caba, Juan Carlos Lopez

**Affiliations:** ^1^University of Castilla-La Mancha, 13071 Ciudad Real, Spain; ^2^Electronics Department, Polytechnic Faculty, University of Mons, Mons, Belgium

## Abstract

In FPGA-based control system design, partial reconfiguration is especially well suited to implement preemptive systems. In real-time systems, the deadline for critical task can compel the preemption of noncritical one. Besides, an asynchronous event can demand immediate attention and, then, force launching a reconfiguration process for high-priority task implementation. If the asynchronous event is previously scheduled, an explicit activation of the reconfiguration process is performed. If the event cannot be previously programmed, such as in dynamically scheduled systems, an implicit activation to the reconfiguration process is demanded. This paper provides a hardware-based approach to explicit and implicit activation of the partial reconfiguration process in dynamically reconfigurable SoCs and includes all the necessary tasks to cope with this issue. Furthermore, the reconfiguration service introduced in this work allows remote invocation of the reconfiguration process and then the remote integration of off-chip components. A model that offers component location transparency is also presented to enhance and facilitate system integration.

## 1. Introduction

FPGAs are used increasingly in control applications. Their processing power, reconfiguration facility, and short design time have made them very suitable for this kind of applications. In addition, some FPGAs offer the ability to reconfigure some of its architecture at runtime without stopping the entire system, which increases their potential for use in control applications, especially for critical systems. In these dynamically reconfigurable FPGA-based systems, the logic area can be divided into several reconfigurable regions where a hardware task, or functional modules constituting a hardware task, can be instantiated. Moreover, the dynamic reconfiguration capability allows the design of multiapplication systems, where different tasks share the same resources during the system life cycle. In the case of tasks that are executed only under certain conditions, these tasks are candidates to be implemented in the reconfigurable area, in order to be instantiated only when required. In this way, it is possible to save resources avoiding components that work sporadically being permanently implemented in the static part of the FPGA. This feature is an added value that can be exploited for preemptive systems.

Preemptive systems are those systems that have the ability to suspend the execution of a running task or process in order to run another task of higher priority, such as real-time systems and control systems. For real-time applications such as control applications, multimedia, medical monitoring, and robotic and automotive control, where the results must be produced within a well predefined interval of time to avoid unwanted consequences, we can consider two types of task operations: periodic operation, for those tasks that have to be executed periodically and finished within a given deadline, and aperiodic operation, for those tasks that are executed in response to asynchronous events.

In case of a periodic operation mode, the task scheduling is predefined offline and therefore predictable. Thus, the scheduling of the reconfiguration process is also well known. On the other side, in aperiodic operation mode, an asynchronous event can produce preemption of an executing task in order to allow the execution of a higher priority one. This swapping of tasks can be done using reconfigurable areas in the FPGA in order to save resources as stated before. This asynchronous event can also be expected to appear within a certain period of time and, in this case, can be scheduled beforehand. For the scheduled scenarios described above, this paper refers to the activation of the reconfiguration process as being an *explicit activation* in response to a specific invocation of the reconfiguration process.

Asynchronous events that cannot be scheduled or require immediate attention imply a more reactive system where the immediate execution of higher priority tasks is mandatory. The replacement of running tasks by higher priority ones must be performed preserving the system integrity, a fundamental condition for fault-tolerant systems. However, assuming that this higher priority task is not yet instantiated on the FPGA, an immediate reconfiguration process must be started. That process is triggered by an out of program invocation of a noninstantiated component, producing what this work refers to as an *implicit activation* of the reconfiguration process.

This paper presents a reconfiguration wizard and a Reconfiguration Engine developed to have mechanisms carrying out the explicit and implicit activations of the partial reconfiguration process in a safe and efficient manner. Besides, this work also describes how these activations are heard when invoked from outside the system through what this work simply refers to as *remote activation* of the reconfiguration process.

*Reconfiguration Process Description*. One of the main advantages of real-time systems design using FPGAs is related to the fact that the processes should not compete for processing time, because they are inherently parallel, although they may compete for area or the use of specific resources (BRAM, DSP), especially when shared. Moreover, the dynamic reconfiguration capability of FPGAs facilitates resources distribution, reduces resources demand, and increases the use of shared resources over the system life cycle assuming the following: (i) a negligible cost of context change and (ii) the independence of the tasks composing the system. Therefore, the dynamic reconfiguration process must be carried out in accordance with the execution scenario.

This work aims to overcome some aspects of preemptive systems by focusing on the reconfiguration process of three specific scenarios:

- there is enough space in the reconfigurable region to place, replicate, or relocate a component;
- there is not enough free logical resources and it becomes necessary to remove some components;
- the available space is fragmented, and the relocation of some components will be necessary for it to be consolidated.

The instantiation of the higher priority task is, in the first scenario, straightforward and can be done either by launching an explicit (*scheduled*) or implicit (*out of program*) activation of the reconfiguration process. Moreover, the reconfiguration request might be simple and followed by a single step of immediate reconfiguration.

In the second scenario, it is necessary to choose which component should be evicted based on one or multiple selection criteria, for example, the amount of area needed, the criticality of the evicted task, or the dependency relation between tasks to be swapped. The selection is performed by a scheduler, referred to here as *dynamic scheduler*, and the reconfiguration process may be much more complex, involving several steps to perform, in order to meet the selection criteria. For explicit activation, the reconfiguration pattern, as well as the choice of the component and its location, is completely solved beforehand, whereas, for implicit activation, the selection must be made on-the-fly taking into account the aforementioned requirements, so that the reconfiguration process is done properly. So, for hard real-time systems with strong deadlines, the dynamic scheduler should choose between components deployed in reconfigurable regions attending the preemption request. Hence, in addition to the previous criteria, the selection is carried out by evaluating a cost function that prioritizes components nearing task completion. The development of the dynamic scheduler is out of the scope of this work.

Notice that when the preemption request is received, if the component to be evicted must keep track of its state, the actual state must also be saved for subsequent component reinsertions. This implies a mechanism to stop the execution of processes in a safe manner, saving the state without loss of data consistency and subsequent state recovery. This work refers to as the *persistence* mechanism. The state of the component to be saved includes current values of properties and attributes. These can be, for instance, the current states of the FSM (finite state machine) that controls the execution of a thread, the coefficient values of an FIR filter, or the keys of an encrypt-decrypt process. The values are constantly changing, so the execution must be stopped at a specific moment without losing information that could prevent state recovery. The designer defines these specific moments in the execution path, which this work refers to as *consistency points*, as well as the amount and type of data to be preserved, along with the storage and recovery processes. All this will have an impact on the selection process, because the dynamic scheduler must assess how far the execution is from the nearest consistency point and how long the swapping process will take.

The third scenario contemplates the relocation of components, optimizing the available space by diminishing the fragmented area through components grouping. Components relocation is performed by the Reconfiguration Engine following the instructions of the dynamic scheduler, which has already defined the movements required to perform successful components reorganization.

An important partial reconfiguration requirement is that components sharing the same reconfigurable region must all have the same interface towards the static part of the FPGA. However, this prevents the placement of the component in a region other than the one it was originally assigned to. Therefore, to allow component migration, between regions into which it can fit, means having regions with the same reconfigurable interface. This feature promotes a distributed object paradigm in a reconfigurable system on chip.

Dynamic partial reconfiguration is also vital in systems based on components memory map. In these systems, the initial platform components were allocated to memory addresses at design time. However, components reallocated to reconfigurable regions can be dynamically mapped to new memory addresses. This way, scalability and robustness are granted on-the-fly by the deployment and update of components when required. The mechanism referred to as *location mechanism* provides location transparency, allowing components to be deployed and reached without prior knowledge of the real location address. Besides, this location mechanism allows task migration and replacement, regardless of their nature (HW or SW), thus improving fault tolerance increasing system reliability.

This paper provides a hardware-based approach for the explicit, implicit, and remote activation of processes in dynamically scalable SoCs and a location mechanism to cope with the dynamic integration of new components. The paper structure is as follows: after this introduction section, the next section presents a summary of related works.  Section 3 postulates dynamic reconfiguration based on the object oriented paradigm as well as proposing a model for dynamic reconfiguration for preemptive systems design; the next section details the mechanisms for explicit, implicit, and remote activation of the reconfiguration process; later, the preemption overhead evaluation is presented; the concepts exposed will be illustrated by several use cases in the results section; finally, this work will close with the conclusions section.

## 2. Related Works

The ideas presented in this paper complement previous works on partial reconfiguration process management [1]. Most of the partial reconfiguration management approaches found in the literature show that hardware task-switching flexibility is performed explicitly and the reconfiguration process is previously scheduled to handle the reconfiguration latency (e.g., during the execution of another task using temporal partitioning [2] or by using prefetching techniques [3–5] where the reconfiguration process starts before needed).

An approach to reconfiguration on demand for fully programmable devices was introduced in [6] with a note of applicability for partially reconfigurable devices. Another approach can be found in [7], where a technique for hardware dynamic reconfiguration management based on a runtime reconfiguration manager is presented. This manager implements a Dynamic Hardware Multiplexing algorithm, enabling an adaptive spatial scheduling of tasks on a given reconfigurable architecture. The algorithm is based on two mapping procedures that dynamically relocate the tasks optimizing the use of processing resources according to the workload.

In [8], a methodology for incorporating preemption constrains to multitask VLSI systems is presented. In this work, the context switch cost and performance degradation are analyzed and a proposal to reduce both is presented. This approach does not use dynamic reconfiguration techniques to address the problem.

Related to dynamic planning of reconfiguration of running tasks in a system, the approach introduced in [9] swaps software (SW) and hardware (HW) versions of the same task. This increases system flexibility, since a HW task might be swapped for its SW version, or vice versa, depending on environmental demands. The basis of this approach lies on assigning three different states to the partially reconfigurable regions: free, ready, or busy. The disadvantage of this solution is the fact that it behaves according to a data flow graph defined at compilation time. As a consequence, the system cannot be adapted to an uncontrolled scenario such as those referred to in this paper. As an alternative, [10] proposes an HW scheduler for managing reconfigurable systems at runtime, based on Directed Acyclic Graphs (DAG). The authors understand the reconfigurable region as a set of independent reconfigurable units (RU), without any relationship between them. These RUs might operate simultaneously, providing a multitasking environment. However, this idea of separate RUs could limit the number of solutions because it requires design RUs adapted to the size of the largest module that could be hosted. On the other hand, despite the fact that the authors evaluated their solution with several experiments, they do not provide the mechanism responsible for manipulating the reconfigurable bitstreams. With regard to the latter, [11] proposes VAPRES, a framework for managing partial reconfiguration with a dynamic resource manager that provides both planning and placement of tasks. Their scheduling algorithm follows an offline methodology, since the scheduler behaves according to a predefined and well-known data stream. This fact may limit its usability in varying environments.

The solutions presented are based on the scheduling and management of the partial reconfiguration of components without providing support for preemption, in the sense of managing issues such as stopping and resuming a task in a safe and effective way, as well as task state management.

Task preemption is analyzed in several papers. In [12, 13], a way to extract and restore the execution state of a task in reconfigurable logic is described. State extraction can be obtained by rereading the bitstream of an FPGA to separate the state bits from the configuration ones. State information bits are those that contain all the information about current values of registers and internal memory of the FPGA. Keeping this information is mandatory to recover the last state when the task resumes. However, the extraction of that information, called FPGA read back, is a process that takes time (it requires parsing the entire bitstream of the FPGA). A method using the read back methodology is proposed in [14]. Another approach can be found in [15] where a hardware preemptive multitasking mechanism which uses scan-path register structure is proposed. In [16], a model for task relocation between HW tasks running in partially reconfigurable devices and SW tasks running in a CPU is presented. Each HW task has a local memory divided into working memory area and data or state area. The HW state area is a mirror of the SW state area found in system memory; the same global variables are allocated at the same relative addresses for both, SW and HW state areas. This approach extends the OS to enable relocation and uses synchronization mechanisms.

In [17], a hardware checkpointing method, defined as a technique to store the state of a hardware task during a free faults operation, is presented. In this paper, three different alternatives of checkpointing are presented. The first one is based on memory maps where the state is mapped into memory by a processor. The second alternative is based on circular shift registers, to store the states, and FSM, which controls the position of the shift register as well as the read/write operations. The third model is based on duplicating registers of hardware modules to obtain one clock cycle state copy. The state is composed of the hardware module's internal registers and memory values. In the first two cases, a large penalty is paid in time, because it is necessary to store the state whenever a checkpoint is reached to allow recovery of the last stored state when a failure occurs (rollback process). Whenever a state storing process is made, a time overhead is added to the system decreasing the frequency of the operation. In the third model, a penalty of resources' duplication is paid. Besides that, the model presented is based on the task model approach and the amount of elements defining the state and the needed resources can be large and expensive.

In heterogeneous contexts where persistence is needed, not only for HW task replacement in dynamically reconfigurable areas but also for SW task migration, homogenization of the state of the SW/HW task is necessary. Indeed, this homogenization allows moving the state of a task running in an Instruction Set Processor (ISP) to its HW equivalent in FPGA. In [18], the operating system (OS) for reconfigurable computing (OS4RC) is responsible for reprogramming the task running in an ISP to reconfigurable hardware. The task state information is transferred to the OS when the task reaches what the author calls a switching point, equivalent to the checkpoint in the works cited above. All relevant state information is then transferred by the OS to a secondary heterogeneous processor to resume the task execution. In [19] wrappers are proposed to adapt HW components forming a HW task composed of the component itself (HwIP), the interface, and the wrapper, to adapt the interface to a peripheral bus. The wrapper has a context buffer to store the task context. A hardware design, with either of the proposed wrappers, can thus be swapped out of the partially reconfigurable logic at runtime in some intermediate state of computation and then swapped in when required to continue from that state. The context data is saved to the wrapper context buffer at interruptible states, and then the wrapper takes care of saving the hardware context to communication memory through a peripheral bus and later restoring the hardware context after the design is swapped in. There are no details about implementation of the mode of state transfer.

The work presented in this paper uses for component state persistence and migration the mechanism presented in [20]. This mechanism, which will be described in the next section, is an extension of the reconfiguration service based on distributed object paradigm.

## 3. Middleware-Based Partial Reconfiguration Management Model

Tasks will often need to access shared resources, so proper protocols for the arbitration of the access to shared resources are needed.

A design consists in a collection of tasks mapped in dedicated resources into the FPGA. Depending on the level of granularity and on its complexity, a task can be seen as a simple component or can be formed by several of them. Once the selection of the tasks that will be part of the static portion of design has been decided, the rest of the FPGA will be divided into several reconfigurable regions. Tasks designed to be swapped in or out of the same reconfigurable region must have the same interface towards the static area, in order to avoid inconsistency in signals connecting static and reconfigurable areas. This is a condition not easy to satisfy and affects the mobility of components or tasks between reconfigurable areas. Moreover, when components are designed by third parties (IP), the adaptability process can be hard and prone to error. One way to minimize these problems is to think of the SoC as a distributed system, where the communication infrastructure can be a bus or a NoC and where each hardware or software component inside the SoC can be considered as a distributed object [21–23]. This requires having a layer (middleware) that facilitates the integration of all resources that form part of the SoC, providing them with a unified interconnection model.

**Figure 1:**
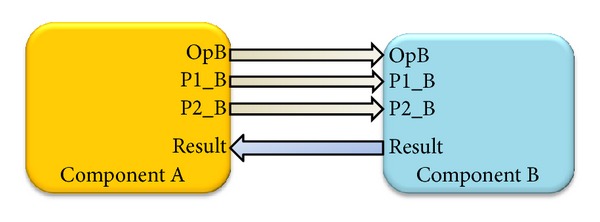
Point to point object communication.

For those components connected point to point, the connection can be represented as depicted in Figure 1, but for those components remotely placed, it is necessary to have a mechanism that allows sending a remote invocation.

**Figure 2:**
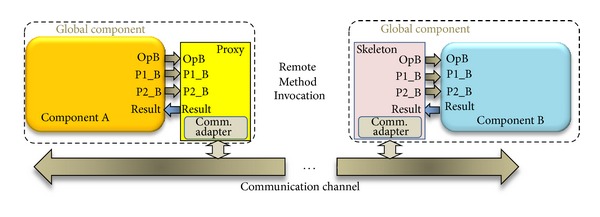
Hardware object adaptation for Remote Method Invocation.

The model used in this work is based on the OOCE middleware [24], where the communication between components is performed by using Remote Method Invocation. The OOCE middleware facilitates component adaptability and communication between objects using the client-server approach. For each client component, a proxy is added that represents the server component (Figure 2). If the client requires services from different servers, a proxy for each one will be added to the client. Each proxy has the same interface as the corresponding server. The function of the proxies is to translate the invocation sent to the servers into messages through the communication channel. In turn, a skeleton is added to each servant component, in order to translate messages coming from the communication channel, into invocations to the server as it was a local invocation. This approach allows three degrees of transparency: (a) location transparency because the client sees the server interface as if it was a local invocation, (b) access transparency because it is possible to reach the server independently of its implementation (hardware or software), and (c) communication transparency because this middleware can be used to adapt any communication channel. Once the communication channel has been defined the design of hardware component adapters (proxies and skeletons) can be done automatically starting from simple component interfaces description.

Hardware components adaptation is depicted in Figure 2. In this figure, an example of component A invoking, through the proxy, a method or operation of component B (OpB) that uses P1_B and P2_B as parameters, is depicted. On the component B side, the skeleton transports the invocation to the server. The adapters are added to each component core and the global component (component core plus adapter) interface is the communication channel interface. This interface will be the same for all components connected to the same communication channel including those instantiated in partially reconfigurable regions (PRR). This is an important characteristic because it allows component or task mobility between different partially reconfigurable areas.

### 3.1. Preemptive Components

To make a preemptive component, it is necessary to add some functionalities to the skeleton to deal with the transference of component state to memory for later reuse. State management inevitably implies that components must be designed for persistence. The component must be able to keep the state when a stop request is received and must be able to send the state for storage if required. Then, the core has to be designed using an introspection interface in order to be capable of self-reading and writing the internal state and to serialize it to/from the skeleton. Persistence management also implies knowledge about types and amounts of data to be stored.

Preemptive components have different skeletons from those that do not support it. This is because the component state management involves handling operations other than those that correspond to the functionality of the component itself. These extra operations are *sizeState*,* getState*,* setState*, and *initState*, which are invoked to know the state size, to store the state in memory, to load the state into the component, and to initialize the state, respectively.

**Figure 3:**
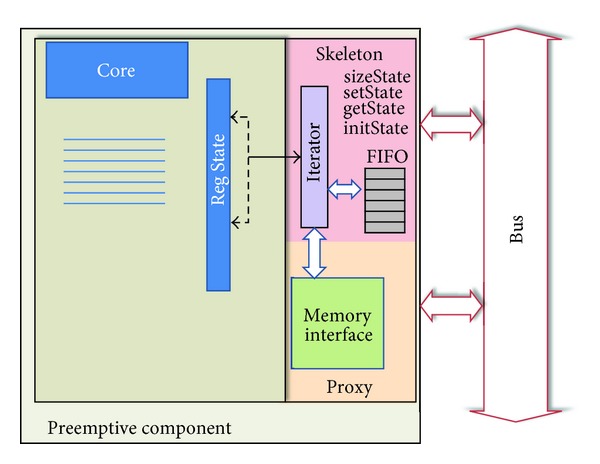
State management of preemptive components.

To deal with these extra operations, an iterator, a FIFO, and a proxy to memory are added to the skeleton as described in Figure 3.

The iterator is responsible for reading the component state, storing it in the FIFO, and sending it to memory in blocks or sequences depending on its size using WriteBlock or WriteSeq methods, respectively. Besides these methods, the iterator has the ReadBlock and ReadSeq methods that are invoked during the recover state process from memory, to read blocks or sequences of data, respectively. As in object oriented paradigm, the iterator is a design pattern used to go through a container and access the container's elements. In the approach presented here, this container is a register (the register Reg State in Figure 3) that is updated with the internal state of the component. Once the component is stopped, the register is updated with the internal state, and then the iterator starts traversing the register to send its content to the FIFO on the skeleton. Once the FIFO is full, or there are no more elements to store, its content is sent to memory through the corresponding proxy. In Section 6, the time consumed transferring the state measured in clock cycles is evaluated. In case of the reinsertion of a component with state, it is necessary to invoke the *getState* method with the memory address of the component state as parameter. Whit this address, the iterator starts moving data from memory to the component.

### 3.2. Dynamic Reconfiguration Process

This work proposes a dynamic resource manager (DRM) structured in layers, where the objective of the DRM is to provide a set of services to perform efficient control of the sequence and processes related to the partial reconfiguration of FPGAs.

The execution of dynamic reconfigurations in FPGAs is typically solved using software approaches, where an embedded processor (such as the MicroBlaze) is used to transfer the reconfigurable bitstream to the configuration memory of the device. As an alternative, the placement responsibility presented in this paper relies on a specialized hardware component (the Reconfiguration Engine) to carry on with reconfiguration tasks directly related to the bitstream manipulation. This approach offers two main advantages: first it represents a two orders of magnitude improvement in partial reconfiguration speed, and, second, no processor is required for the reconfiguration process management.

The top layer of the DRM is formed by this Reconfiguration Engine which is responsible for the dynamic placement (and deployment) of the reconfigurable components on the reconfigurable grid. Most of these tasks are completely device and technology dependent and, for that reason, the main purpose of the Reconfiguration Engine is to provide a common abstraction of the technology involved in the reconfiguration process, encapsulating this mechanism as a transparent service to the upper layers. At the bottom layer of the DRM, the reconfigurable region is configured as a grid of composable reconfigurable areas. A minimum reconfigurable microarea unit is defined for the specific system, which can be used separately, or integrated into a larger reconfigurable macroarea at runtime. In this context, the DRM is able to manage this dynamic 2D area model of the FPGA, where the reconfigurable region is structured as a homogeneous grid in which several areas might be coupled into bigger areas at runtime. This characteristic of agglutination makes the reconfigurable region more efficient since areas can be adapted to match with reconfigurable modules of different shapes and sizes (Figure 5(b)).

**Figure 4:**
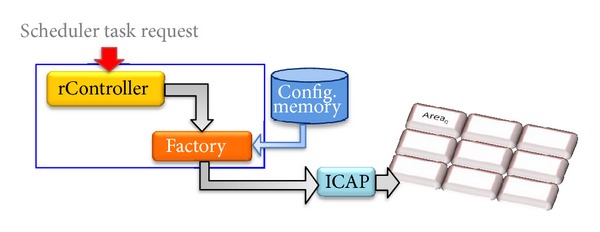
Dynamic reconfiguration manager.

The Reconfiguration Engine offers a set of reconfiguration services through a simplified interface. Those services include bitstream transference, the start and stop of individual instances, status requests, and location and relocation of components. From the architecture point of view, the Reconfiguration Engine is composed of the Reconfiguration Controller (rController) and the Factory. It also requires some kind of storage resources, such an external memory (see Figure 4), although it is not part of the Reconfiguration Engine itself. In addition, it includes an internal table that dynamically stores the state of the reconfigurable fabric: location of the instances, bitstream references, and the execution status of the instances, or memory references for module persistence issues.

#### 3.2.1. Component Location Service

Having the possibility to add some new objects after the system has been deployed offers a solution in pre-emptive systems that allows the system to keep running, especially when a failure in a component is found. System breakdown can be avoided by adding a new component during runtime to replace the bad component, improving in this way system fault tolerance. If the system cannot be stopped, adding new hardware objects to the systems requires that these objects must be instantiated in partially reconfigurable regions of the FPGA. Once implemented each one of these new objects must be univocally identified in order to be reachable for the rest of objects in the system. All designed objects, implemented, either in software or in hardware (Static HW Objects or Dynamic HW Objects depending whether if they are implemented in static or reconfigurable area, resp.), have a unique ObjectID (object identifier) formed by the modID (the module identifier, where a module is, in terms of object oriented paradigm, the class defining the object) and the instID which represents the identifier of each one of the implemented component instances. In those bus-based systems, each component has associated a unique reference inside the system memory map formed by the aforementioned ObjectID. In network-based systems, the ObjectID forms part of the message used to communicate processes to identify source and destiny.

As mentioned before, one of the characteristics of the system-level middleware is that it provides different types of transparency in the use of resources, such as location and access transparency which allows clients to invoke methods of a remote object without any prior knowledge of either their real location or type of implementation. In its simplest form, location transparency is obtained providing the physical references (ObjectID) of the objects inside the corresponding proxies and skeletons.

The model of managing reconfiguration process presented in this paper has a very important characteristic that allows the incorporation and the management of new components after system deployment. Let us suppose that it is necessary to include a new component into the system, to replace a bad one, or to add new functionality. In order to reach this new component, it is necessary to have a location mechanism that provides the component location (address) to the requesting clients.

This mechanism that we call Component Location Service is provided by two entities: the proxy and the Locator. The proxy stores two references, the physical reference of the server that represent, and a reference to the Locator. The Locator, on the other side provides the valid address from the requested object identity. The Locator contains a location table where each objectID is associated with a valid reference point (object address). To add new components, it is necessary first to register the new ObjectID and its corresponding reference point, or address, in the location table. When the Locator receives an invocation of the locate method with the ObjectID as parameter, the Locator returns the valid object address to the proxy.

Let us evaluate the case of adding a new version of an already instantiated object to the system. If this new version replaces the previous one, then it will use the same ObjectID and the same address of the previous version so the invocation process does not change. But let's suppose now that the designer wants to keep both versions in the same system, so the new version will have a new ObjectID and will require a new address. If a client needs services from a server that was not predicted at design time, its proxy should be changed with the new server ObjectID.

Let's suppose now that a component is assigned to a new address. Figure 4 describes the typical flow of indirect location. First, the client requests from the server a certain operation. Then, the proxy forwards this request to the last known memory address of the server (the location of the server). (1) If this address is no longer valid, a timeout error will arise, so the proxy will cancel the request. (2) Next, the proxy invokes the Locator with the identity of the server object to be contacted and stores the returned memory address as the new last known address. (3) Finally, the proxy issues the request again, this time to the valid new memory address (4).

It may seem that indirection implies certain communication overhead and extra invocation latency, due to the extra messages to the Locator. However, this indirection occurs only once and then, with the new endpoint assigned, the server is localized for further invocations. This location mechanism increases the fault tolerance and the adaptability of the system to changing environments.

Besides, this location mechanism provides the basement for the implicit reconfiguration activation model: when a noninstantiated component is invoked and the request is resent to the Locator, as previously described, the Locator can start a reconfiguration process if the component is registered but not instantiated.

## 4. Reconfiguration Activation Models

As mentioned before, this approach provides three ways of activation of the reconfiguration process: (a) explicit activation occurs when the reconfiguration process is explicitly demanded by a component or application, (b) implicit activation, where the reconfiguration process is triggered out of program, due to the invocation of a nonavailable object, and (c) remote reconfiguration, which is launched by an off-chip component or application.

The reconfiguration assistant is responsible for the reconfiguration process that includes the following steps: (1) to send the stop request to the candidate object to be evicted or disabled, (2) to send the request to store the object state, (3) to send to the Factory the request to partially reconfigure the FPGA, (4) to send the request to load the state of the new instantiated object, and (5) to activate the new object. To accomplish all these functions, the reconfiguration assistant uses an internal table where all objects are registered. This table, called KnownObjects Table, associates each ObjectID with (a) the corresponding one or more reconfigurable areas where the object can fit, (b) the system memory map address (object address), and (c) the memory reference in which the object state is to be stored.

The reconfiguration assistant performs three main functions: (1) allocate a new object into the partially reconfigurable region (PRR), (2) reallocate a known object into a PRR, and (3) locate an existent object inside system memory map. In order to partially reconfigure the FPGA, the allocate method must be invoked if the object to be instantiated is new, or the reallocate method if the object was previously instantiated. On the other hand, if the locate method is invoked the assistant looks up for the object address in its KnownObjects Table. One of the main advantages of this approach is that this reconfiguration service allows explicit and implicit partial reconfiguration process activation. In the explicit version, only an invocation to the corresponding method is needed. In the latter, if a method invocation of a noninstantiated but registered object is performed, implicit reconfiguration process activation is triggered, which will place the required object into the corresponding PRR for later use. This feature allows dynamically adding new objects to the system, first registering them into the reconfiguration assistant and then activating them through methods invocation.

### 4.1. Implicit Activation Process

The implicit activation process occurs when a noninstantiated object is invoked. The detailed process is shown in Figure 6. It is worth recalling that the objects depicted in the figure can be implemented either as hardware or software component. When object A invokes object B, this invocation is sent to the last registered address (step 1). If object B does not answer, a timeout error is sent through the communication channel. Then the proxy of object B invokes the locate method of the rController (step 2). If the object is registered in the rController's KnownObjects Table, but it is not instantiated, the reconfiguration process takes place. If the requested object is already instantiated, then an indirection process occurs as indicated in Section 3.2.1. Inside the reconfigurable area an object C may already be running. If so, a stop invocation is sent to it (step 3). After the object C was stopped and its state saved, the rController invokes the Factory to start the reconfiguration process. The Factory takes the corresponding bitstream from memory and reconfigures the corresponding area through the ICAP (step 4). The object B comes on stage. Finally, the object is activated, the address updating is performed (step 5 in Figure 6(b)), and now the object B is ready to be invoked (step 6 in Figure 6(b)).

If object B is not registered, an error message will be sent to the proxy through the Locator. With this error message, the object A will stop the invocations to object B and will send an exception message to the scheduler.

When an external event occurs and demands immediate attention, the response of this event can be triggered by means of an interruption requested by the external component or using polling to the external inputs. In both cases, once the external event had been detected, the scheduler according to the priorities of running objects, invoke the Rengine to start reconfiguration process instantiating the requested component. The development presented here allows redirecting the external invocation directly to the Rengine which after verification, if requested component is registered, starts the reconfiguration process. This alternative has the limitation that it is necessary previously to define in which area the requested component will be instantiated. If the requested component can be placed in several places the responsibility to choose the best reconfiguration scenario falls on the planner. In the predefined reconfiguration scenario case, the process is as described above.

### 4.2. Remote Activation

Communication, though, is not restricted to the SoC internals. In a computational distributed system, the communication between components placed in different SoCs is frequent and method invocation of internal SoC objects can arrive from outside the chip (off-chip communication).

If the execution of certain tasks that include partial reconfiguration is required, then it might be of interest to the remote activation of the reconfiguration process. In order to cope with this requirement, the OOCE middleware includes a device to facilitate off-chip communication. This device called Remote Network Interface (RNI) adapts different protocols such as Ethernet or RS232 to the system communication channel.  Figure 7 shows the block diagram of the RNI. The RNI, from the logical point of view, provides a bridge between two different middlewares, a commercial one and the internal system middleware OOCE. The RNI is the component that guarantees the interoperability between these two worlds (on-chip and off-chip). The mission of the *ICE-Bridge* component consists in processing and creating messages, in the external middleware format, from and to the outside the chip, respectively. In this case, the external middleware used is ICE (Internet Communication Engine) [25], a CORBA-like software commercial middleware. When an external message arrives, the Remote Object Adapter (ROA) component of the RNI has the responsibility to determine if the invocation is read or write invocation that will be launched into the SoC. If the invocation has a return value, the ROA will be in charge of the reception of the value and will send it to the *ICE-Bridge* in order to be sent outside the chip. The adapter translates internal addresses into object and method names, encapsulates internal messages in, for example, UDP, and routes them to the network addresses. These addresses have to be previously registered in the adapter's internal table.

## 5. Preemption Overhead Evaluation

Each preemption causes an increase in the runtime overhead due to the operations executed during a context switch. In this section the relevance of this overhead is analysed. As established in [26], each task Ti is characterized by a worst case execution time (WCET) Ci, a relative deadline Di, and a minimum interarrival time It.

The task set shown in Figure 8 corresponds to a system assuming constrained deadline model in which corresponding Ci, Di, and It are indicated in Table 1. This system results to be unschedulable by Deadline Monotonic [27], both in fully preemptive and in fully nonpreemptive mode, but it can be schedulable by all limited preemptive approaches [26]. For hardware tasks preemption, the schedule produced by Deadline Monotonic can be solved assigning different reconfigurable areas for each task forming a system with tasks running in parallel. In case that different priority tasks share resources, the lower priority task can migrate to a new area (free or occupied by also a lower priority task) and continue its execution when preempted.

Let's suppose that tasks *τ*_2_ and *τ*_3_ from Figure 9 share the same area and *τ*_2_ is the task with higher priority than *τ*_3_. Assuming that *τ*_2_ can be deployed only in a specific reconfigurable region and *τ*_3_ is already implemented in this region, *τ*_3_ should be stopped and migrate to another place or to another implementation or just stopped and its state saved until *τ*_2_ is finished, depending on the corresponding deadline. In this case, the WCET should include not only the execution time but also the migration time if the lower priority task will continue its execution in an other reconfigurable region or in software.

Therefore, the runtime overhead caused by context switching should be added to the WCET forming the Extended Worst Case Execution Time (EWCET). This EWCET in case of deadline constrained systems has to be less than the interarrival time It and the relative deadline of the corresponding task. In the same way, the time consumed in context switching must be considered to determine the feasibility of task relocation before tasks switching.

This work considers preemption overhead as the time consumed during the whole preemption process.

To evaluate the preemption overhead introduced by this approach several aspects need to be taken into account. To reconfigure an area with an already instantiated object in there, it is necessary to perform three main steps: to stop the low-priority task and to save its state, to reconfigure the area, and to load the state of the high-priority task. Each one of these steps will be evaluated as follows.

  (1) The stop of the low-priority task starts sending a stop invocation to the task. The time elapsed since the stop invocation was sent until the state transfer confirmation was received is formed by the following components:
  (1) $$\begin{array}{c} T_{\text{stop}\_\text{DRO}}=T_{\text{stop}\_\text{meth}}+T_{\text{ack}}+T_{\text{state}\_\text{trf}}. \end{array}$$
 This time includes the following.
  (i) *T*_stop_meth_ represents the time consumed to stop running methods in the object to be evicted and depends on if the object is either active or passive as discussed previously. For active ones *T*_stop_meth_ will depends on the stage of the execution path of the corresponding method, and in case of passive objects, *T*_stop_meth_ is the time consumed to finish the method in execution;
  (ii) *T*_ack_ indicates the time elapsed while the acknowledgment of stop invocation is received by the rController;
  (iii) *T*_state_trf_ represents time necessary for state transference from object to memory. This time is characterized by
    (2) Tstate_trf=StSizeChwidth∗Ncycles+init_time,
 where St_Size_ indicates size of state to transfer in words of 32 bits, Ch_width_ indicates communication channel width (in words), and *N*_cycles_ is the amount of clock cycles for each communication channel transaction. Init_time indicates initialization time required to transfer the state from core to the FIFO.
 (2)  Once the object was successfully stopped and the state stored, the next step is to perform the partial reconfiguration of the FPGA. The time consumed during partial reconfiguration process (*T*_pr_) depends on the size of the bitstream, the memory access time, and the speed of internal reconfiguration of the FPGA given by the ICAP.
 In the next Section different values of *T*_pr_ using the approach introduced in this paper will be presented.
 (3) Once the new DRO is implemented the next step is to evaluate the time needed to load its state and to activate it. This time will be defined mostly by the consumed time loading object state (if any), represented in Table 2, because to send an activation object invocation it takes just a couple of clock cycles.
 Therefore, the total time forming the preemption overhead is the sum of the time involved in the three mentioned steps (see (1) and (2)):
  (3) TPO=Tstop_DRO+Tpr+Tstate_trf.
 The last operand of (3) is equal to 0 if the new component has no state.
 Finally, the EWCET can be estimated by means:
  (4) EWCET=WCET+TPO+2xTpr,
 in which the last term corresponds to the two partial reconfigurations performed to instantiate two times the lower priority task.

### 5.1. Implicit Reconfiguration

The latency introduced when an implicit activation of reconfiguration is produced was also evaluated. This latency is formed by the following parameters. Once obtained state transference time (*T*_state_trf_) and reconfiguration time (*T*_pr_), the next step is to analyse the delay introduced by using the implicit reconfiguration mechanism;

(5) Timpl_rec=Terr⁡+TRCloc_meth+TPO,

where

- *T*_impl_rec_ is the overhead introduced by implicit activation mechanism;
- *T*_*err*⁡_ is the time consumed during timeout error;
- *T*_RCloc_meth_ is the time elapsed during invocation of the locate method of the Rengine.

## 6. Experimental Result

The experiments performed to evaluate conceptually and to measure the preemption overhead of the approach presented in this paper are based on an indoor localisation and orientation distributed service built on a dynamically reconfigurable platform [28]. Indoor navigation, life assistance, disaster preventions, proximity marketing, security concerns, personal localisation, multitude monitoring, and augmented reality are examples of services that require a localization and orientation service. Analyzing the video streaming sent by the users through their smart phones or tablets in the environments where users are, it is possible to extract their position and orientation with several degrees of accuracy as a function of the analysis algorithm.

The algorithms used in this application field have two common characteristics: first, they require a previous exhaustive characterization of the environment, which generates a large database through the extraction of a set of points of interest (corners, windows, etc.) that are used to match against those extracted from the images taken by the user. The general idea is to find those points of interest in the users' video streaming to infer their position/orientation. The other characteristic in common is the computer power requirements of these algorithms to perform an efficient video analysis and an exact comparison with the database of the environment.

The Features from Accelerated Segment Test (FAST) and Corner Detection Algorithm [29, 30] allow the detection of features points (corners) in frames from a parameterizable set of surrounding pixels. The FAST algorithm is used as a part of a mechanism of localization and orientation in indoor environments using Parallel Tracking and Multiple Mapping (PTAMM) methods [31]. This method performs the tracking of common features separately between maps and the mapping process. The tracking method uses the information provided by the current map to find a camera pose for the new frame that is being evaluated.

The PTAMM builds a map of the scene, matching objects that are observed as matched features in images taken from different points. Simultaneously, it uses this map and the current image to make an estimation of the camera pose. During the initialisation phase, PTAMM builds an initial map using two frames of the same scene taken from two different points and separated by a known distance to set the scale of the map. Once both images have been captured, Faugeras and Lustmans' method [32] is used to compute a new camera pose and to triangulate scene points [33]. FAST is used to detect feature points in the first frame and these points will be searched for in all incoming frames. These images, called keyframes, are processed by FAST to detect features that will be used to match with new keyframes.

In order to accelerate the detection of feature points, two different implementations of the FAST algorithm performed in hardware were selected to be deployed in reconfigurable areas. These hardware-based FAST modules (HW-FAST) were developed so that they can determine a corner from a parameterizable set of surrounding pixels without incremental computational time. In this work, each pixel is evaluated in one clock cycle without the need to perform compression on the patterns and without implementation of a decision tree [29, 30] because the comparison is performed for all pixels at the same time.

Figure 10 depicts the block diagram of the system developed to evaluate the approach presented in this paper.

Once users are logged into the system they can make use of the service sending a video streaming from their portable device to the server. The service provider sends the video streaming to the FPGA for processing. The image is first converted to 8-bit data (gray-scale image) and then is sent to the FPGA through the Ethernet port. The Control Component separates the payload of the message and sends the pixels and the HW-FAST configuration data such as the threshold and the amount of pixels to calculate the feature point. This new frame is the input of the HW-FAST component, which after work sends back a file with the coordinates of each characteristic point. The Packet-Mux component is the component that creates each file of coordinates when both HW-FAST components are working simultaneously and creates the data packets to be sent through the Ethernet channel. With these coordinates, the server recomposes the image, adding these points. With these points added to the map, in the mapping phase of the PTAMM methods, the location is identified and the Augmented Reality components are added to the image to compose the localization and orientation service output.

**Figure 5:**
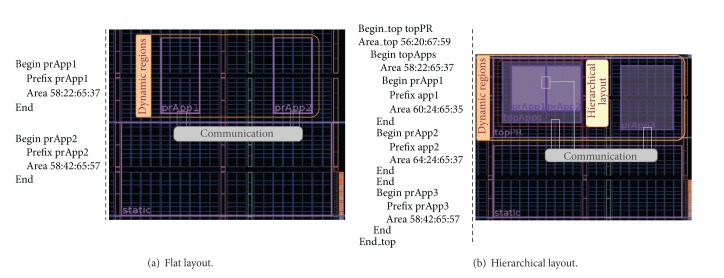
Dynamic reconfigurable areas layout.

**Figure 6:**
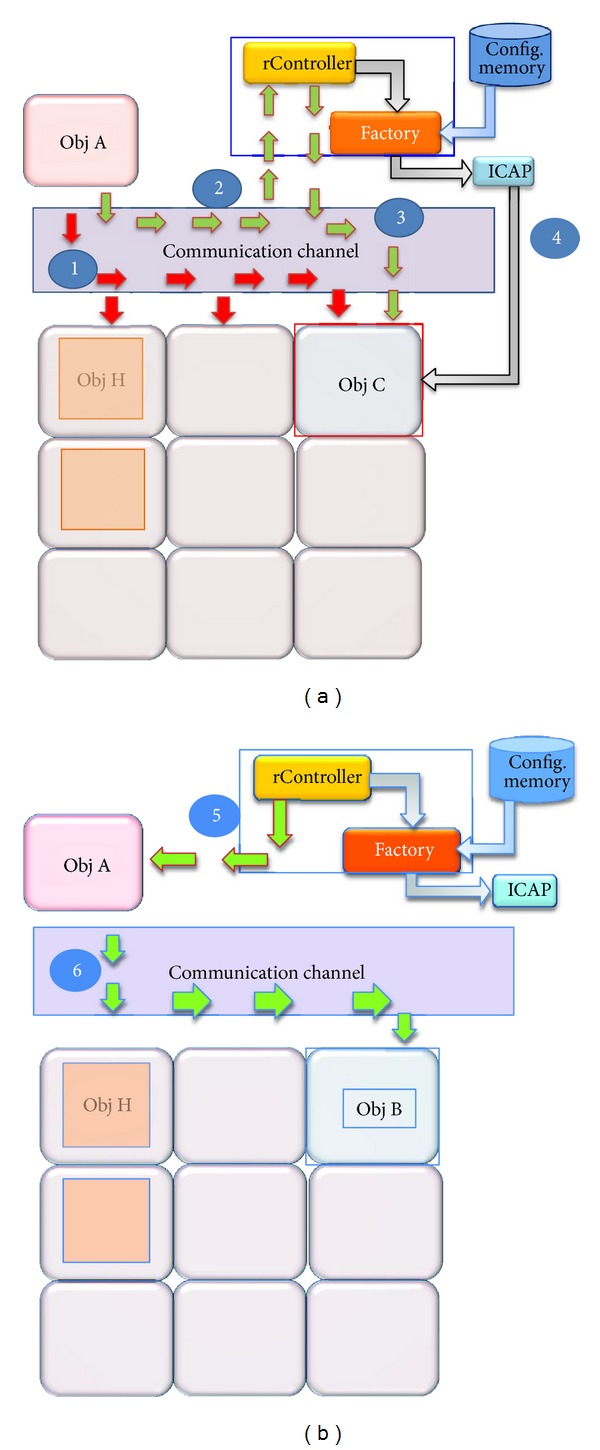
Implicit reconfiguration process.

**Figure 7:**
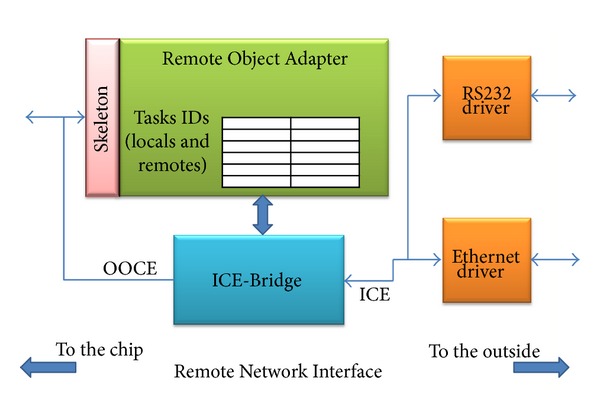
Remote Network Interface.

**Figure 8:**
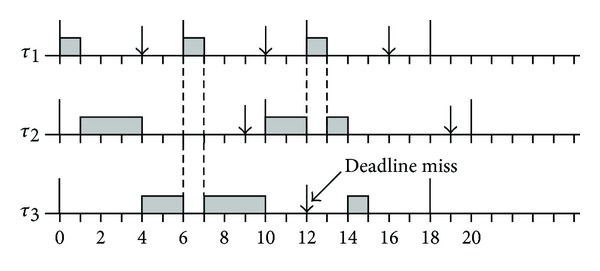
Schedule produced by Deadline Monotonic in fully preemptive mode.

The HW-FAST components and the Control and Packet-Mux components have been prototyped over the Xilinx Virtex 5 and Virtex 6 FPGA-based boards. For these tests, several reconfigurable areas have been defined using hierarchical layout, as represented in Figure 5(b).

In areas prApp1 and prApp2 were implemented two HW-FAST components running the FAST algorithm for corner detection. These two implementations allow serving two clients simultaneously. The system is designed in such a way that allows the implementation of more HW-FAST cores depending on the demand implementing more HW-FAST components in reconfigurable areas when required.

The implementation of new HW-FAST components can require the eviction of some task running in the target area. For a conceptual proof of the approach presented in this work and to measure the time involved in the preemption overhead, a publicly available DES code was implemented in prApp2 that will be evicted when a new HW-FAST is required.

The behavior of the DES is described as follows: the data to be encrypted is stored in a memory block. A couple of extra iterators were used to go through this block and, after data encryption, to store the encrypted data back to memory. In this experiment, the object state is formed by the encryption key and two memory pointers, one for the original block and the other for the encrypted data block. The regular status of the system is with one HW-FST running in area prApp1 and a DES running in prApp2. Each time the number of logged users into the system requires an extra HW-FAST, the reconfiguration mechanism is triggered and the DES component is stopped and prApp2 is reconfigured instantiating a new HW-FAST component inside prApp2.

### 6.1. Preemption Overhead Evaluation

As previously stated the preemption overhead is formed by (1) the stop of the low-priority task and its state saving process, (2) the reconfiguration of the area, and (3) the load of the state of the high-priority task.

#### 6.1.1. Stop Task and State Transference

In our example, to stop the DES component it takes 2 clock cycles.

To test this proposal for state transference several dynamic objects of different sizes were evaluated. The FIFO width was 32 bits in the implementation, and three transference ranges were evaluated:

- StateSize = 1 word;
- 1 word < StateSize < FIFO size;
- StateSize = FIFO size.

In all cases the evaluation was in terms of clock cycles/word consumed during the transference to and from memory. Two kinds of storage elements were used for estimation: (a) local memory and (b) external DDR memory. For the last type of memory two kinds of transferences were performed: one using single writes and reads and the other using bursts. The results are summarized in Table 2. This table shows the consumed time (measured in clock cycles) to save the state as a function of state size. When the state size is greater than 1 word, there is an initial time to be considered (the init column), that is, the time consumed filling the FIFO which depends on the FIFO depth. In the case presented the depth was equal to 8 words taking 2 clock cycles per word to fill the FIFO. In this table, it can be observed as well that the delay for state extraction or reinsertion is very low, roughly a few clock cycles. The first row indicates the time that it takes to send the component state to the internal memory of the FPGA (Block RAM) while the second and third rows indicate the time that it takes to send the state to an external DDR memory word by word or using burst, respectively.

The time consumed storing DES state, according to (2) and considering a size state of 4 words of 32 bits (the encryption key and the two memory pointers), is without using burst

(6) $$\begin{array}{c} T_{\text{state}\_\text{trf}}=4\times 33+8=140\;\text{clock}\;\text{cycles}. \end{array}$$

Therefore, using (1),

(7) Tstop_DRO=142  clock  cycles,

which for a frequency of operation of 100 MHz represents 1.42  *μ*seconds.

#### 6.1.2. Partial Reconfiguration Time

The reconfiguration speed achieved using the Rengine described in Section 3.2 is about 180 MB/s. It is quite straight to observe that reconfiguration times obtained using the Rengine improve the results presented in [34] and [35]. This improvement is due to the use of the full bandwidth of the ICAP and the parallel execution of the configuration management tasks.

For the experiments presented in this paper, the sizes of the bitstream corresponding to each component in prApp3 are indicated below:

 HW-FAST.bit = 342631 Bytes,
 DES.bit = 523195 Bytes.

Then, taking into account the reconfiguration speed, the time *T*_pr_ consumed in reconfiguring these bitstreams is:

(8) Tpr=1.90351 ms for  HW-FAST  component,

(9) Tpr=2.90664 ms for  DES  component.

#### 6.1.3. Loading State of High-Priority Task

Once the HW-FAST was reconfigured in area prApp3, the next step is to transfer its state from memory. The state is formed by the pointer to memory to extract the pixels of the frame and the 49 bytes corresponding to the ring of pixels being analyzed. If the preemption mechanism is triggered by a request of a new HW-FAST, initially this component does not need to load state. So there is no time consumption in loading initial state.

Therefore, the total Preemption overhead is then (according to (3), (7), and (8))

(10) TPO=0.00142+1.90351=1.90492  milliseconds.

**Figure 9:**
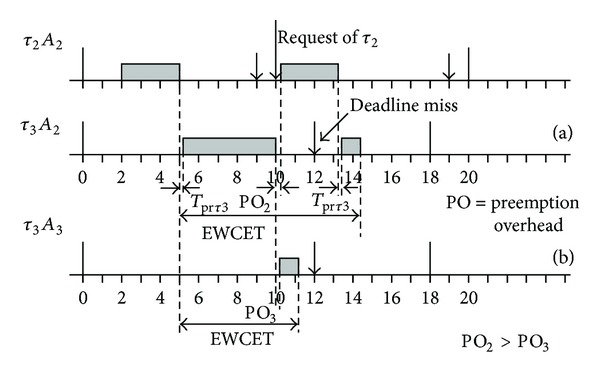
Schedule produced by dynamic reconfiguration in fully preemptive mode.

**Figure 10:**
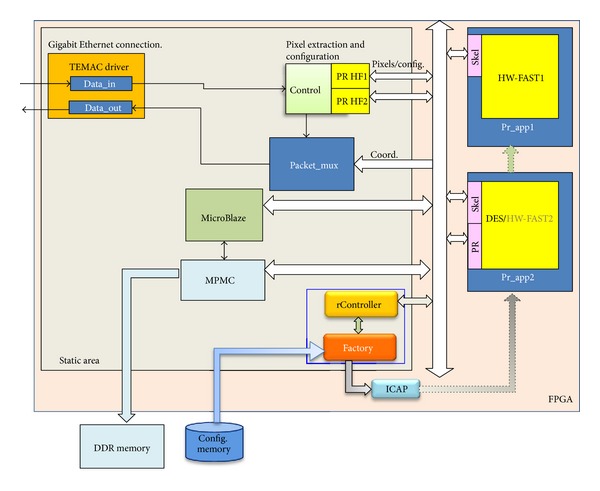
Block diagram of HW-FAST implementation.

Finally, the EWCET (see Figure 9) of task DES can be calculated from (3), (4), (9), and (10) as

(11) EWCET=1.90492+2.90664×2+WCET,EWCET=WCET+7.71820  milliseconds.

Then, the EWCET for the DES task can be obtained adding the preemption overhead (7,7182 milliseconds in this example) that is basically the partial reconfiguration time, to the WCET defined by the scheduler.

As it can be seen, the preemption overhead is mostly produced by the reconfiguration time of the tasks sharing the same area: first, the reconfiguration time of the less priority task, then the reconfiguration time of the new task, and finally the reconfiguration time of the first one again when reinserted. Therefore, the preemption overhead is directly related to the size of the bitstreams to be loaded and can be perfectly characterized to facilitate the scheduling of reconfiguration process.

### 6.2. Implicit Reconfiguration Latency

To test the overhead introduced by the implicit activation mechanism described in this paper, several experiments redirecting invocation to a valid endpoint were performed. The communication overhead introduced by the indirection process can be divided into two parts: the overhead introduced until the error timeout occurs (16 clock cycles for PLB bus) and the proxy endpoint updating. The latter, measured in terms of clock cycles, takes between 5 and 8 clock cycles after the reconfiguration process ends. This communication overhead occurs only once. Once updated, the proxy will send the subsequent invocations to the correct endpoint.

### 6.3. Remote Reconfiguration Invocation

To evaluate the latency introduced when a remote invocation of reconfiguration is performed, a completely hardware implemented RNI was used. The RNI connects the system-level middleware to an Ethernet network.

In this work, the RNI component does not use connection-oriented communication like TCP because their control in hardware increases the complexity of the core. Two-way communications are not supported by this block, but they are translated into two one-way invocations.

Furthermore, the RNI translates local messages to the ICeP [25] protocol used by ICE. Messages are routed between ICE and OOCE using UDP, so complete interoperability between both middlewares is achieved for one-way invocations. Remote invocations to the reconfiguration assistant allocate method were performed to start the reconfiguration process using RS232 driver and Ethernet. For the RS232, the time elapsed since the external invocation to the reconfiguration process was sent until the invocation is in the bus was 80 *μ*seconds (rate 115000 bauds).

For the Ethernet network, the delay of an external invocation through the RNI took 332 clock cycles. This delay includes the time elapsed from the UPD message reception inside the core Ethernet of the testing board, up to the acknowledgment from the reconfiguration assistant injected into the bus. The size of Ethernet frame used was 85 bytes. For this case, the endpoint updating from the rController to the Ethernet device took 200 clock cycles. The clock frequency for the RNI was 100 MHz, and then the overhead introduced by the RNI was around 5.3 *μ*seconds.

## 7. Conclusions

In this paper, partial reconfiguration techniques were presented to facilitate preemptive system design. Mechanisms for implicit and explicit partial reconfiguration process activation were described. Also a component designed to manage off-chip communication adapting different communication protocols was presented to facilitate the remote activation of the reconfiguration process. Besides a location mechanism was presented to provide components location transparency. The reconfiguration technique presented in this paper based on distributed object paradigm was created to allow the integration of new components and the mobility of components already between different reconfigurable areas, obtaining more versatile designs and improving fault tolerance. A mechanism for state management in preemptive components was also presented and the preemption overhead introduced by this proposal was characterized and evaluated. The combination of location transparency of components (the capacity to invoke any object—inside or outside the chip—as it was a local invocation), access transparency (the capacity to reach any object independently of its implementation), and explicit, implicit, and remote activation of the reconfiguration process modes provides great benefits in the overall system design and deployment process, allowing the incorporation of new objects at runtime not considered during the initial stages of the design. In particular, flexibility of execution is achieved by supporting task migration and upgrading hardware with new features to prioritize different computational modes according to the priority of tasks, a challenge that can be now addressed with the solution presented in this paper.

## Figures and Tables

**Table 1:** Example tasks set.

Task	Ci	Di	It
τ_1_	1	4	6
τ_2_	3	8	9
τ_3_	6	12	18

**Table 2:** Time consumed in state transference (*T*_state_trf_) measured in clock cycles per word.

	32-bit word		State size = FIFO depth		
	Store	Load	Init	Store	Load
Internal memory	7	7	16	2	3
External memory	10	42	16	33	40
External memory (burst)	—	—	16	2	2
